# Factors affecting school enrolment and attendance for children with disabilities in Bangladesh: Evidence from a cross-sectional survey

**DOI:** 10.1371/journal.pone.0309402

**Published:** 2024-10-24

**Authors:** Shilpi Rani Saha, Soumik Saha

**Affiliations:** 1 Bangladesh University of Professionals, Mirpur Cantonment, Dhaka, Bangladesh; 2 Scholastica, Dhaka, Bangladesh; Austin College, UNITED STATES OF AMERICA

## Abstract

In Bangladesh, many disabled children are not attending school. Despite the country’s special policies, laws, and services, most disabled children face a barrier to enrollment in school. To increase the school attendance of children, particularly disabled children, research on barriers to school attendance is essential. It will help to ensure that all children, including those with disabilities, receive an education. This paper will investigate the factors associated with children with disabilities missing school. This study will utilize data from a large sample based on Nationally representative multiple indicator cluster survey 2019 in Bangladesh. The study group included 5024 disabled children ranging in age from 6 to 17 years. To determine the significant covariates for the multivariate analysis, a bivariate analysis was performed. The findings indicate that age of the child, gender, household head sex, education of the household head, area (urban/rural), husband age, whether the husband has more wives, and disability types are all associated with disabled children being absent from school. Using these findings, the government of Bangladesh and other stakeholders should advocate for policies and programs that reduce barriers to education and ensure that all disabled children attend school.

## Introduction

More than 1.6 billion children from all over the world attend school on any given day. More children and adolescents than ever before are enrolling in pre-primary, primary, and secondary school. However, 11% of primary-school-aged children and 20% of lower-secondary-school-aged children are not enrolled in any school at all [1, 2]. Children and adolescents are denied access to education for a variety of reasons, disability is one of the most common reasons for children missing school [2]. Children with disabilities face greater barriers to attending and finishing school in developing countries [1]. While legislative efforts to promote school access for children with disabilities have received a lot of attention, there is a lack of empirical research to base policy on. The lack of research is largely due to a lack of relevant and comparative data [1].

Internationally, studies have shown varying factors influencing school attendance for disabled children. For instance, research in India [3] and Kenya [4] highlight socioeconomic status, parental education, and infrastructure as key determinants. Comparatively, in Bangladesh, factors such as rural-urban disparities and household head education levels are significant. Understanding these international and regional differences provides valuable context for addressing the unique challenges faced in Bangladesh.

It is extremely difficult to collect quality data for disabled children due to the various definitions and measures of disability. It is difficult to estimate the number of children with disabilities and the types of challenges they face without good quality data [5]. The 2019 Multiple Indicator Cluster Survey (MICS) data are of high quality for assessing the situation of Bangladesh’s children, adolescents, women, and households [6].

As a result, the primary goal of this study is to evaluate the associated factors for out-of-school children in Bangladesh using MICS 2019 data. Many of the fundamental factors influencing childhood disability and schooling remain unknown and unaddressed. To contribute to policy development, this paper will investigate the interaction between children with disabilities and those who are not in school.

We reviewed relevant studies to highlight the independent variables commonly used and provided a comparative perspective on global and regional trends in school access for disabled children. This includes factors such as age, gender, household characteristics, and disability type.

## Children with disabilities

Basically, there is no widely accepted definition of the term "disability." Furthermore, it varies according to its nature and severity [7]. "Disability is defined as a situation that limits an individual’s physical, mental, or physical development [8]." However, the appropriate definition of disability is determined by the purpose for which it is measured. Disabilities are still primarily measured medically [7]. According to the medical model of disability, disability is a medical condition that affects an individual. The social model of disability, on the other hand, believes that disability is the result of a person’s functional state interacting with their physical, cultural, and policy settings [7]. According to the World Health Organization’s (WHO) International Classification of Functioning (ICF) framework, "disability is an umbrella term referring to activity limitation, participation restrictions, and impairments [7]." The United Nation’s Convention defines disabilities in such a way that people with disabilities are those who have long-term physical, mental, intellectual, or sensory impairments that, when combined with other hurdles, prevent them from achieving their full potential. Furthermore, because disability is a state of reduced functioning caused by a disease, accident, or other health condition, it is viewed in the context of one’s environment as an impairment, activity limitation, or participation restriction [9]. To allow people with disabilities to fully participate in society, social attitudes and government regulations must change [8].

Quantitative research on out-of-school for disabled children is hampered by differences in the definitions of "disability". In our study, we defined disability based on functional difficulties as reported by caregivers, aligning with the Washington Group on Disability Statistics (WGDS). This definition, focusing on limitations in activities and participation, differs from other studies such as the one using Household Income Expenditure Survey (HIES) 2010 data, which may use a broader or more medical-oriented definition. Understanding these differences is crucial for interpreting the outcomes and comparing results across studies [10]. According to the Persons with Disability Welfare Act, 2001 in Bangladesh, disability is defined as a physical, mental, visual, or intellectual impairment that prevents a person from engaging in normal day-to-day activities. Disability is defined as the inability to live a normal life without the assistance of others. As a result of their situation, they rely on family, community, and the state for attention, aid, and collaboration [10, 11]. Children with disabilities have become a major public health concern around the world. Globally, in 2017, the approximate number of children with disabilities was 291.2 million, which was about 11.2% of the total 2.6 billion children and adolescents. The majority of them are from low- and middle-income countries [12]. The situation in Bangladesh is considerably worse. Disability prevalence in Bangladesh ranges from 1.4 percent to 17.5 percent, depending on the definition of disability and the method of data collection [13]. In 1982, following the International Year of Disabled Persons in 1981, the Bangladeshi government began conducting disability surveys, and the disability rate was 0.64% [13–15]. According to the World Bank (2004), 3.4 million children in Bangladesh have a disability, accounting for 6% of the total population [11]. As per the Bangladesh Population and Housing Census (2011), 8.0 to 10 million children have some kind of disability, out of a total of 57.5 million children [13]. On the other hand, several newspaper reports imply that the number of disabled children in Bangladesh has been increasing in recent decades. In Bangladesh, 17 children out of every 1000 are born with autism [13]. According to the MICS 2019, the child disability rate was 7.3% among children aged 2 to 17 years old, and the majority of disabled children in Bangladesh were found in rural areas rather than urban areas. 7.6% were born in rural areas, followed by 6.1% in urban areas [6].

Bangladesh is one of the few countries to have disability data from very limited options [13–16]. Greater attention is needed to childhood disability for early intervention, prevention, and rehabilitation [17]. Early intervention is more effective when it includes an active parenting and skill-building policy and provides adequate services directly to children rather than providing information alone. Although knowledge of child development and the nurturing of children among Bangladeshi mothers has increased after information-based sessions, that may have an influence on reducing childhood disability [18]. In Bangladesh, disabled children are suffering from social stigma, ignorance and negligence. That can be the result of social isolation and a deprivation of basic human needs. Although according to the Constitution of the People’s Republic of Bangladesh, every human, including disabled children, has equal rights in all respects, most of the disabled children are frequently exposed to unfriendly, noncooperative, neglect, and mistreatment in society as well as in government institutions [10].

**Fig 1:**
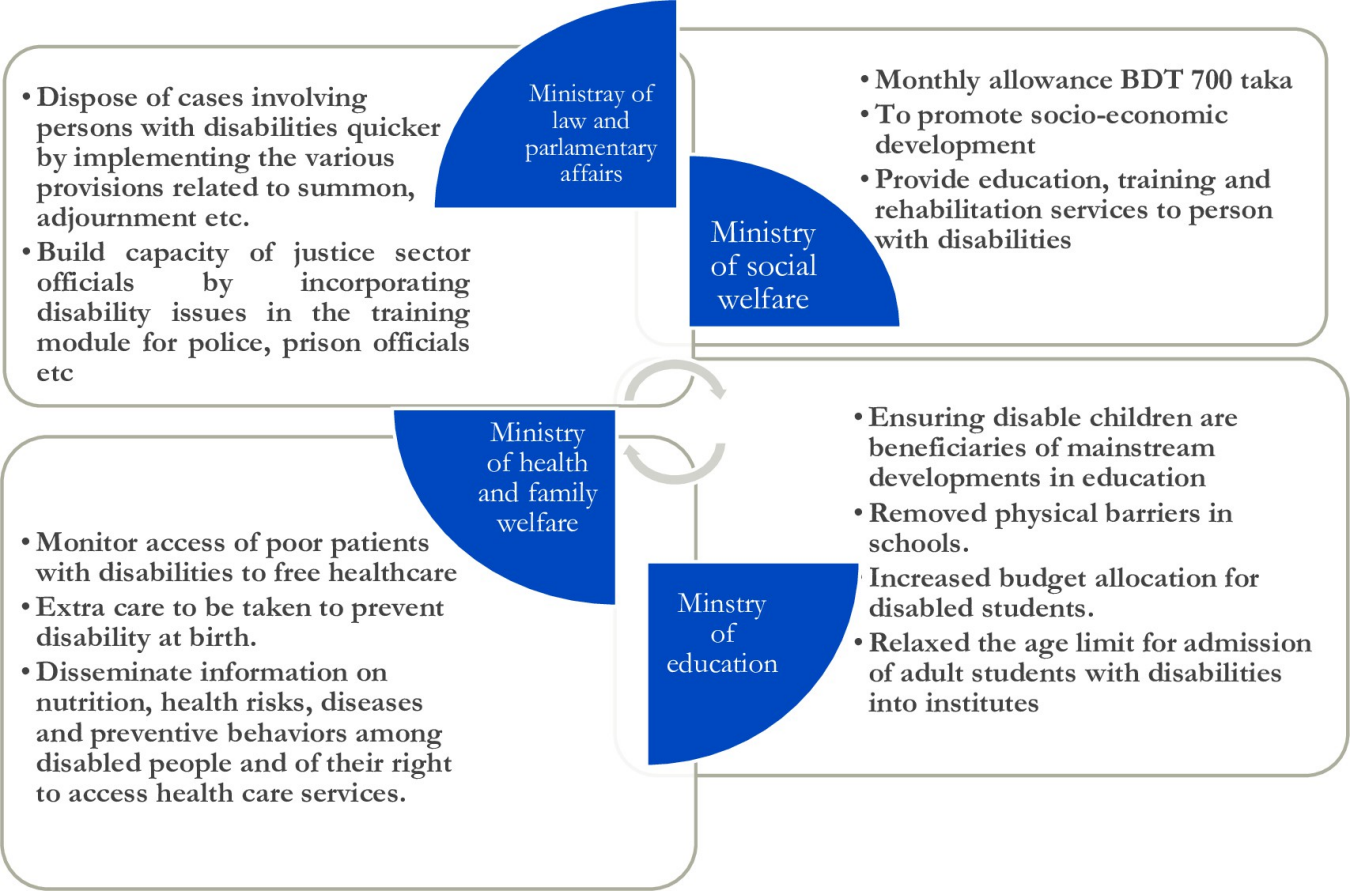
Different ministerial and inter sectoral political commitment to disability [19].

According to the "Protection of the Rights of Persons with Disabilities Act, 2013" in Bangladesh, every disabled child deserves the same freedom and dignity. The main aims of the Act are to ensure the educational, physical, and mental advancement of children with disabilities and to facilitate their inclusion in social and state activities through the removal of all types of discrimination [16]. Bangladesh’s government has taken some political responsibilities to decrease the overall dishonor associated with disability (see Fig 1). However, a large proportion of disabled children are not included in treatment, therapies, or specialized curriculum [19].

The above (Fig 1) showing multi ministerial commitment to disability in Bangladesh.

Despite equal chances for everyone to access public services, disabled children in Bangladesh face complexity in accessing facilities [16]. The convention on the rights of persons with disabilities outlines that a disabled person can be entitled to all types of facilities provided by the government, the same as a non-disabled child [19].

## Education for children with disability in Bangladesh

All children have the right to receive a high-quality basic education. Every child, including those with disabilities in Bangladesh, has constitutional rights to education. Bangladesh has made significant contributions to education in recent years [10]. The Primary Education (Compulsory) Act of 1990 made primary education free and mandatory. "Education for All (EFA)" is a worldwide initiative and popular slogan that includes people with disabilities. Bangladesh has taken a number of steps to improve access to EFA indicators and gender equity. As a result, the net primary enrollment rate improved from 65% in 1997 to 91% in 2007. However, these accomplishments are limited because the country has failed to ensure that all groups of people have equal access to education equally. In particular, children with disabilities are still facing problems with entrance into educational institutions and discrimination [10, 15, 20].

Bangladesh’s educational system consists of five years of compulsory primary education for all children, including children with disabilities, three years of junior secondary school, two years of secondary school, and two years of higher secondary school. Education is only compulsory and free at the primary level, with girls receiving free education up to the secondary level [15]. Bangladesh’s National Education Policy (Ministry of Education 2010) was adopted in 2010 to address the present educational agenda, which included inclusive education [20]. Article 28 (3) of Bangladesh’s constitution states clearly that no citizen should be subjected to any disability, liability, restriction, or condition solely on the basis of religion, race, caste, sex, or place of birth with regard to admittance to any public place of entertainment or resort, or to any educational institution [10, 20]. In addition to its legal requirements, the government is committed to integrating disabled students into school [11].

Due to a lack of a solid database and survey in Bangladesh, important statistics on the disabled and their educational position are insufficient and unreliable. Various studies found that between 75 and 77 percent of disabled children were not in school [10]. People’s negative attitudes, community invisibility, educational cost, physical access, class size, lack of skilled teachers, gender discrimination, and identification of children with disabilities are all considered the main barriers for school enrollment for disabled children in Bangladesh [10, 11, 14]. Moreover, education and skill development opportunities for children with disabilities have yet to be developed or created in the country, and the situation remains unsatisfactory. As a result, children with disabilities frequently face various challenges in acquiring an education [10]. In order to overcome this situation, the Bangladesh government has taken several initiatives to improve disability education. In addition to its legal requirements, the government has made a strong commitment to implementing mainstreaming education for disabled students. The government opened 13 special elementary schools for children with disabilities in 2011. They are also creating 64 integrated programs for disabled students in high schools. These efforts are definitely having an effect, yet many disabled children may not have access to these settings. There is a clear need to dramatically expand these programs, including the establishment of new disability-focused schools across the country and ensuring that all schools include programs for disabled students [10, 13–14]. Although the country is making significant progress in expanding the enrolment of children with disabilities in mainstreaming schooling as a result of the execution of the Primary Education Development Programme (PEDP) and related complementary measures [15]. However, the large number of children with disabilities who are denied access to primary education represents a significant waste of resources for the country; it challenges the goal of ensuring equitable quality education for all by 2030 on education-related SDGs and other development goals [21].

In Bangladesh the Bangladesh Rural Advancement Committee (BRAC) has been trying to expand education for children with disabilities. There is still absence of significant disability programming in public schools. BRAC is dedicated to ensuring that poor children and those living in remote places have access to education [13, 14].

Overall, government and non-government organizations, such as BRAC, are making progress, but more growth and expansion are needed to ensure that all children with disabilities in the country have access to high-quality education. This will relieve their families’ financial burdens and, perhaps, enable them to find a job once they reach adulthood, allowing them and their families to overcome poverty.

As a result, the overall goal of this study is to identify the factors that contribute to disabled children missing school in Bangladesh. Also, will provide some strategy for ensuring equal access to quality education for all children, including those with disabilities. We strongly believe that the useful information provided by the present study will be helpful for policymakers and researchers in designing inclusive education for children with disabilities, which ultimately allows them to realize their full potential. Furthermore, it is intended that the information will help to achieve the Sustainable Development Goals (SDGs).

## Materials and methods

### Study design

This study used large-scale, nationally representative multiple indicator cluster survey (MICS) 2019 data. The MICS was conducted in collaboration with the Bangladesh Bureau of Statistics (BBS) and the Ministry of Planning from January 19 to June 1, 2019. UNICEF Bangladesh supported the survey with both technical and financial assistance. The MICS 2019 for Bangladesh gathered data on 144 important indicators, 29 of which are directly relevant to the Sustainable Development Goals (SDGs). The MICS is a household survey that delivers data that is nationally representative, accurate, statistically sound, and internationally comparable. MICS data is widely used in policymaking and intervention planning. A representative sample from eight divisions and all 64 districts in Bangladesh’s urban and rural areas is included in the 2019 MICS [6].

This cross-sectional survey applied two-stage stratified cluster sampling. The sample frame of the Population and Housing Census of Bangladesh from 2011 was used to select the cluster. At the first stage, the primary sample unit (PSU) was calculated by using the probability proportional to size (PPS) procedure from the 293,533-census enumeration area. In each sample region, a list of households was conducted, and the sample household was chosen in the second stage. A systematic sample of 20 households was drawn in each of the chosen PSUs after a household listing was conducted within the selected enumeration areas (cluster). Since the published report contains full information on the survey methodology is available on (BBS and UNICEF Bangladesh, 2019) [6].

According to the mother/caregivers, 5024 children aged 6–17 years had any kind of functional difficulty, which is the ultimate sample of our present study. Here we included children’s ages starting from 6 years because in Bangladesh children enter primary school at age 6. Although the government of Bangladesh has mandated one year of pre-primary education since 2010, we focused on children aged 6 and above to align with the primary school entry age, ensuring a consistent and relevant dataset for our analysis. This also aligns with the objectives of investigating factors affecting primary school enrolment and attendance.

The data was collected using five different face-to-face questionnaires. (1) members of the household; (2) younger children (under the age of five); (3) children (aged 5 to 17 years); (4) women (aged 15–49 years); and (5) a questionnaire for household water quality testing [6].

### Measuring childhood disability, outcome variable and independent variables

Since childhood disabilities are one of the major focus areas of this study, additional information about child functioning, including childhood disabilities, is included. We have used MICS 2019 in this study, as it used questions to determine the proportion/number of children with functional difficulties. A child was classified as screening positive if the mother or primary caregiver reported any functional difficulties. In Table 1, questions about disabilities are presented. Functional domains covered for children age 5–17 are as follows: seeing, hearing, walking, self-care, communication, learning, remembering, concentrating, accepting change, controlling behavior, making friends, anxiety, and depression.

**Table 1:** Survey question for child functioning.

Questions list	
1	When wearing (his/her) glasses or contact lenses, does (name) have difficulty seeing?
2	When using (his/her) hearing aid(s), does (name) have difficulty hearing sounds like peoples’ voices or music?
3	Without (his/her) equipment or assistance, does (name) have difficulty walking on level ground?
4	Does (name) have difficulty with self-care such as feeding or dressing (himself/herself)?
5	When (name) speaks, does (he/she) have difficulty being understood by people?
6	Compared with children of the same age, does (name) have difficulty learning things?
7	Compared with children of the same age, does (name) have difficulty remembering things?
8	Does (name) have difficulty concentrating on an activity that (he/she) enjoys doing?
9	Does (name) have difficulty accepting changes in (his/her) routine?
10	Compared with children of the same age, does (name) have difficulty controlling (his/her) behaviour?
11	Does (name) have difficulty making friends?
12	I would like to know how often (name) seems very anxious, nervous or worried.
13	I would also like to know how often (name) seems very sad or depressed.
Answer list	
No difficulty. . . . . . . . . . . . . . . . . . . . . . . . . . . . . . . . . . . . . . . . . . . . . . . . . . . . . . . . . . . . . . . . . . . . . . . . . . . . . . . . . . . . . . . . . . . . . . . . . . . . . . . . . . . . . . . . .1	
Some difficulty. . . . . . . . . . . . . . . . . . . . . . . . . . . . . . . . . . . . . . . . . . . . . . . . . . . . . . . . . . . . . . . . . . . . . . . . . . . . . . . . . . . . . . . . . . . . . . . . . . . . . . . . . . . . .2	
A lot of difficulty. . . . . . . . . . . . . . . . . . . . . . . . . . . . . . . . . . . . . . . . . . . . . . . . . . . . . . . . . . . . . . . . . . . . . . . . . . . . . . . . . . . . . . . . . . . . . . . . . . . . . . . . .3	
Cannot see/ hear/ walk/ care for self/ be understood/ learn things/remember things/	
concentrates/ accept changes/ control behavior/ make friend at all. . . . . . . . . . . . . . . .4	
Answer list for anxiety and depression	
Daily. . . . . . . . . . . . . . . . . . . . . . . . . . . . . . . . . . . . . . . . . . . . . . . . . . . . . . . . . . . . . . . . . . . . . . . . . . . . . . . . . . . . . . . . . . . . . . . . . . . . . . . . . . . . . . . . . . . . . . . . . . . . . . . .1	
Weekly. . . . . . . . . . . . . . . . . . . . . . . . . . . . . . . . . . . . . . . . . . . . . . . . . . . . . . . . . . . . . . . . . . . . . . . . . . . . . . . . . . . . . . . . . . . . . . . . . . . . . . . . . . . . . . . . . . . . . . . . . . . .2	
Monthly. . . . . . . . . . . . . . . . . . . . . . . . . . . . . . . . . . . . . . . . . . . . . . . . . . . . . . . . . . . . . . . . . . . . . . . . . . . . . . . . . . . . . . . . . . . . . . . . . . . . . . . . . . . . . . . . . . . . . . . . . . .3	
A few times a year…. . . . . . . . . . . . . . . . . . . . . . . . . . . . . . . . . . . . . . . . . . . . . . . . . . . . . . . . . . . . . . . . . . . . . . . . . . . . . . . . . . . . . . . . . . . . . . . . . . . . .4	
Never. . . . . . . . . . . . . . . . . . . . . . . . . . . . . . . . . . . . . . . . . . . . . . . . . . . . . . . . . . . . . . . . . . . . . . . . . . . . . . . . . . . . . . . . . . . . . . . . . . . . . . . . . . . . . . . . . . . . . . . . . . . . . . .5	

MICS 2019 introduces a new module to identify children with disabilities based on the Washington Group on Disability Statistics (WGDS: http://www.washingtongroupdisability.com/). The modules are based on children’s difficulties in a variety of domains as reported by mothers or caregivers. The WGDS suggested cut-offs for disability are scoring 3 or 4 on these questions, except for anxiety and depression. 1 is suggested for measuring disability only for anxiety and depression [6]. Because disability-related questions were included in such a large scale, nationally representative survey conducted by UNICEF and BBS, we can use household characteristics in Bangladesh to investigate the factors associated with disabled children’s absence from school.

In order to perform the analyses of out of school children, we used a dummy variable of school participation (1 = currently out of school) and a dependent variable of school attendance (0 = currently attending school).

Independent variables in this study include age (6 to 17 years), and the sex of the child is characterized as either male or female. Average household size includes the number of household members in a household and is divided into three categories: 2–3, 4–5, 6 and above. The total number of children living at home is also divided into three categories: 1–2, 3–4, and 5 and above. Parental or family information is also included for analysis. The sex of the household head is also characterized as either male or female. The education of the household head and mother is categorized as pre-primary or none (less than 1 year), primary (1–5 years completed), secondary (9–10 years of education completed), and higher+ (11+ years of education completed, including professional or university degrees). An area is categorized as urban or rural. The age of the husband is a categorical variable: less or equal to 40, 41 to 50, 51 to 60, 61 years and above. we included “If a husband has more wives” as an independent variable to explore the potential impact of polygamous family structures on disabled children’s school attendance, it is categorized as yes or no. Previous studies have suggested that family dynamics, including polygamy, can influence resource allocation and parental involvement in children’s education. While we excluded the religion indicator due to its minimal variation and significance in preliminary analyses, we acknowledge its importance in other contexts and studies.

At least one disability type is mainly categorized as two types, namely: at least one disability and more disability. The wealth index quintile (poorest, second, middle, fourth, and richest) is considered the socio-economic status of households. The wealth index in the MICS 2019 survey was calculated using principal component analysis of household assets, such as ownership of durable goods (such as televisions and bicycles) and dwelling features (such as the source of drinking water, sanitation facilities, and construction materials) [6]. These weighted values were then added together and rescaled to a range of 0 to 1, with each family being divided into five quintiles: poorest (first quintile); second (second quintile); middle (third quintile); fourth (fourth quintile); and richest (fifth quintile) [MICS 2019].

We selected independent variables based on their relevance to the study objectives and previous research. The religion indicator was excluded due to its limited impact on school attendance in the context of our study.

### Statistical analysis

Simple bivariate and binary logistic regression was used in this study. In this analysis, simple cross-tabulation (chi-square) analysis was done to examine the relationship between the outcome variable and the independent variables. The main advantage of using bivariate analysis (Chi-square test) is that, it’s provides information whether there is a significant relationship between the outcome and the independent variable. At multivariate analysis, a binary logistic regression model was fitted to find out the factors associated with out of school among disable children of 6 to 17 years of age in Bangladesh. Binary logistic regression predicts a dichotomous outcome based on a collection of predictor variables that can be continuous, discrete, dichotomous, or a combination of any of these. SPSS version 26 was used for all statistical analysis.

## Results

**Fig 2:**
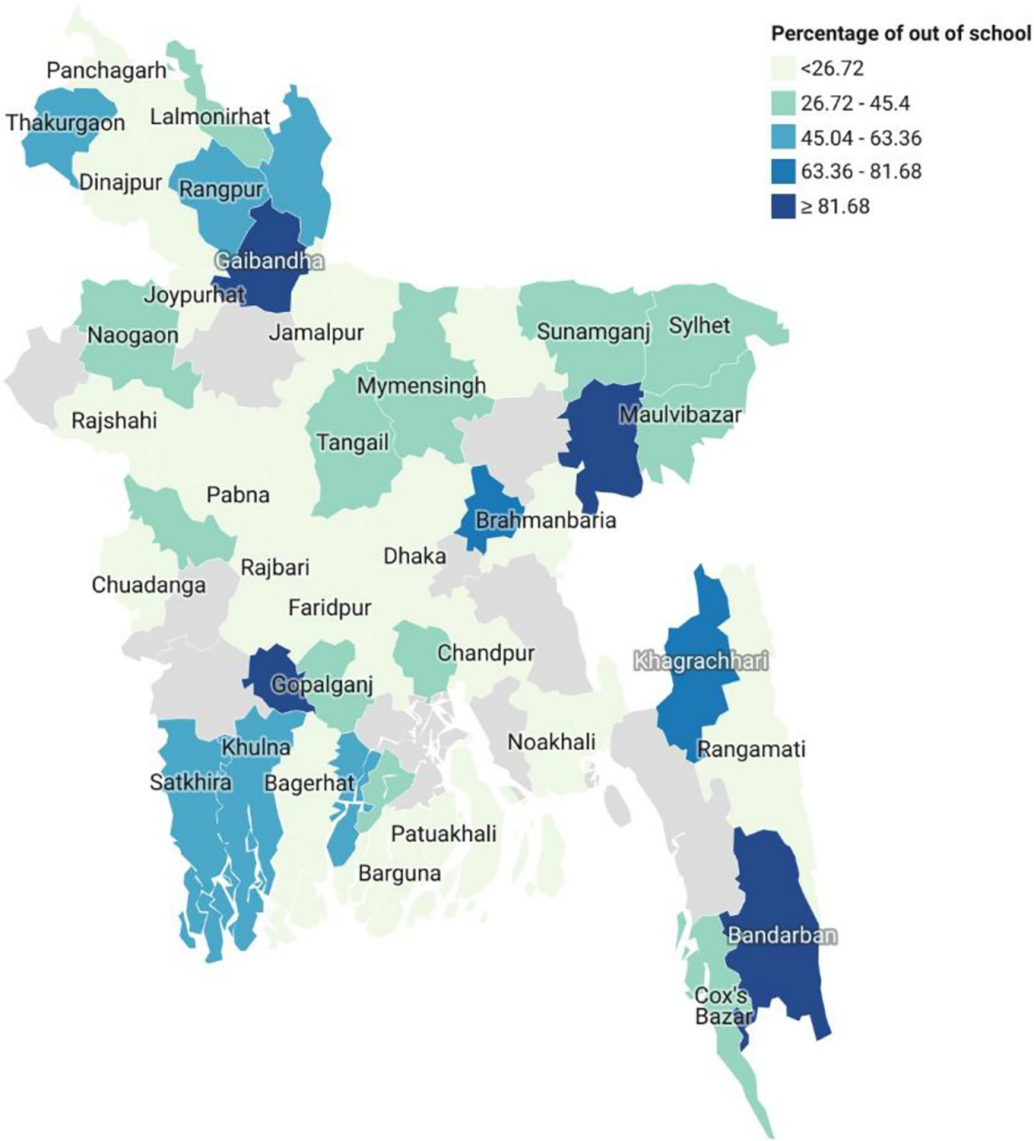
A district-wise mapping on the distribution of out of school of disabled children in Bangladesh.

The district-level spatial mapping of out-of-school disabled children (Fig 2) reveals that out-of-school disabled children are common in most districts in Bangladesh. Within 64 districts, only one district, named Lakshmipur, had 100% disabled children in school. The highest percentage of out of school children is in four districts, namely, Bandarban, Habiganj, Jashore, and Narail, where more than 80% to 100% of disabled children were out of school.

Table 2 represents the school attendance of school-age disabled children by demographic characteristics. Disabled children were considered out of school if they had never attended school or an early education program. This classification includes early education programs to capture the full scope of educational participation, acknowledging that early education is a critical step towards primary education, as highlighted by the USAID report and the UN SDG target 4.2.

**Table 2:** School attendance for disable children aged 6 to 17 years by demographic characteristic.

	Schooling status of Children with disability			
Variable		Disable children in school	Disable children out of school	P value
	6	84.0%	16.0%	0.00
	7	91.6%	8.4%	
	8	88.3%	11.7%	
	9	86.6%	13.4%	
	10	84.6%	15.4%	
	11	82.8%	17.2%	
	12	80.1%	19.9%	
	13	71.1%	28.9%	
	14	67.4%	32.6%	
	15	68.8%	31.2%	
	16	57.7%	42.3%	
	17	44.7%	55.3%	
Sex	Male	73.6%	26.4%	0.00
	Female	82.0%	18.0%	
Average Household size	2–3	75.5%	24.5%	0.063
	4–5	82.7%	17.3%	
	6 and above	73.0%	27.0%	
Total number of children living at home	1–2	78.4%	21.6%	0.003
	3–4	82.6%	17.4%	
	5 and above	41.7%	58.3%	
Sex of household head	Male	81.4%	18.6%	0.001
	Female	66.2%	33.8%	
Education of household head	Pre-primary or none	71.7%	28.3%	0.004
	Primary	82.2%	17.8%	
	Secondary	82.4%	17.6%	
	Higher+	96.9%	3.1%	
Mother’s education	Pre-primary or none	65.6%	34.4%	0.000
	Primary	78.1%	21.9%	
	Secondary	84.0%	16.0%	
	Higher secondary+	93.5%	6.5%	
Area	Urban	80.0%	20.0%	0.036
	Rural	76.9%	23.1%	
Age of the husband	Less or equal to 40	90.4%	9.6%	0.000
	41 to 50	83.4%	16.6%	
	51 to 60	64.5%	35.5%	
	61 years and above	71.4%	28.6%	
Husband has more wives	Yes	62.5%	37.5%	0.016
	No	80.9%	19.1%	
Disability type	At least one disability	86.2%	13.8%	0.000
	More disability	70.1%	29.9%	
Wealth index quintile	Poorest	73.1%	26.9%	0.000
	Second	71.2%	28.8%	
	Middle	80.5%	19.5%	
	Fourth	80.4%	19.6%	
	Richest	87.7%	12.3%	

Disable children out of school means if the response was "no" to either of the two questions: Ever attended school or an early childhood program? during the current school year, attended school or an early childhood program Table 2 shows that school participation for the disabled is higher for those below age 13. The out of school rate is higher among male (26.4%) than female (18.0%) children. If the total number of children living at home was 5 and above, the out of school (58.3%) rate was significantly higher among them. Table 2 also shows that the out of school rate was significantly higher among those whose household head and mother’s educational status was pre-primary or uneducated, i.e., 28.3% and 34.4%. Rural disabled children were less likely to have educational attainment than urban children. 23.1% of rural children were out of school as compared to 20% of urban children. Out of school is significantly higher if the husband is older than 50 years and has more wives. A greater proportion of disabled children were out of school (29.9%) who had more disabilities compared to those with at least one disability. The out of school rate was higher among those disabled children whose families were poor.

**Fig 3:**
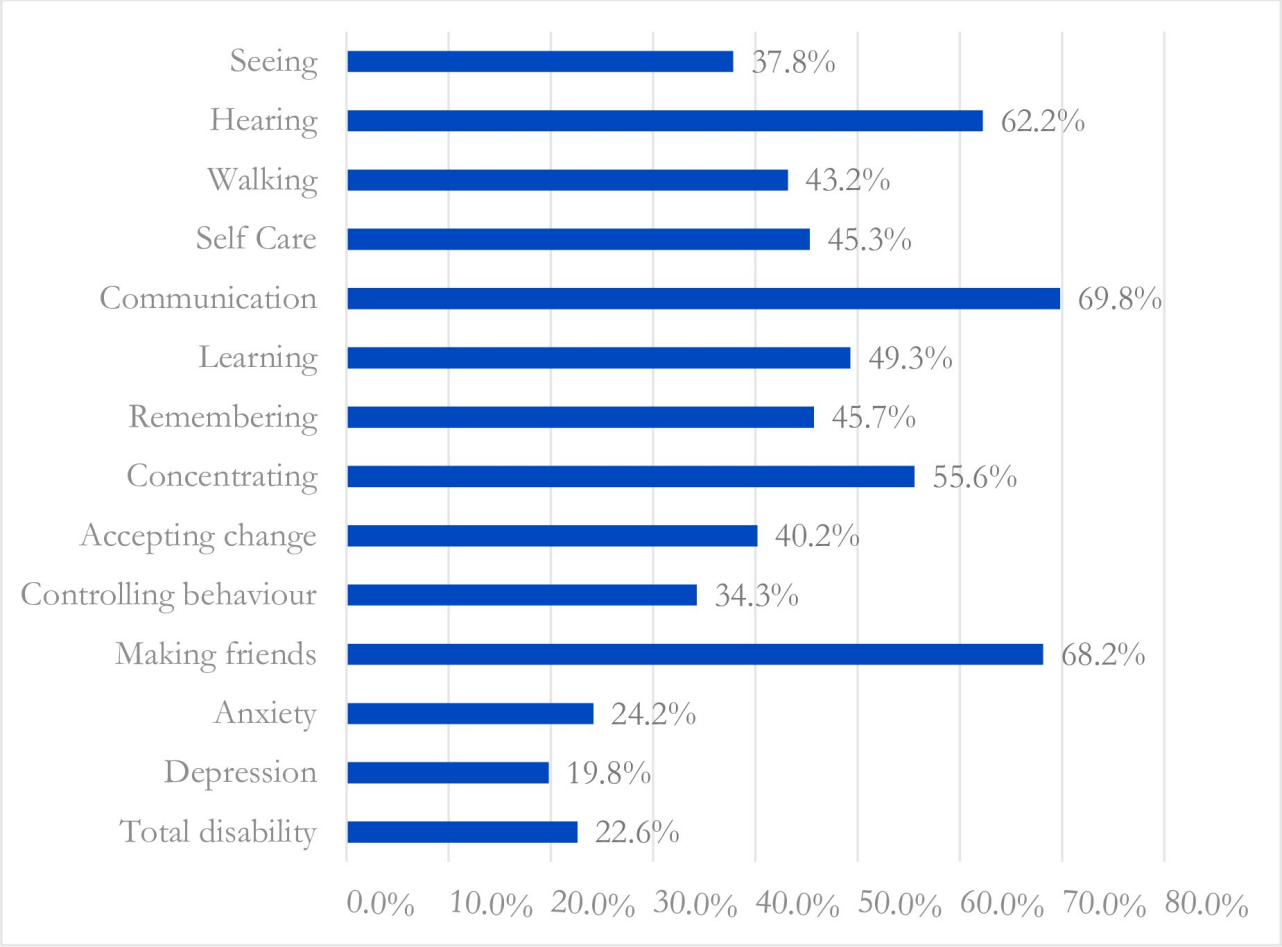
School age (6–17 years) children with disability out of school rate by types of disability.

Different types of disability also impact children’s school participation rate. Fig 3 represents the out of school rate of disabled children by their types of disability. The out of school rate was higher among those children who faced difficulty in hearing, communicating, and making friends.

Table 3 shows the binary logistic regression results for determining the associated factors of disabled children’s access to education. Significant factors (P 0.05) include age of the children, sex, household head sex, education of household head, area, husband’s age, and disability types. It is evident that each one-year increase in age is associated with 1.34 times (OR = 1.34, CI 95% 1.18–1.52, *p* value 0.00) increases in time out of school for disabled children. The empirical findings of the study suggest that disabled male children were more likely to be out of school than disabled female children (OR = 8.47, CI 95% 4.06–17.67, *p* value 0.00). The out of school rate was higher among those children whose household head was female as compared to male (OR = 4.70, CI 95% 1.76–12.52, *p* value 0.00). Disabled children from uneducated households have a significantly higher dropout rate (OR = 4.29, CI 95% 1.91–9.57, p value 0.00 Rural disabled children had a higher probability of being out of school. The odds of being out of school were 0.07 times higher in rural areas compared to urban areas, indicating a greater barrier to education access in rural settings (OR = 0.07, CI 95% 0.02–0.18, *p* value 0.001). The results show that the age of the husband (51 years and above) had a significantly higher probability of being out of school of disabled children (OR = 2.64, CI 95% 1.37–5.12, *p* value 0.001) than those where the husband’s age is less than or equal to 50. Children of the family where the husband had more wives were about 3.57 times more likely to be out of school compared to the children’s family where the husband had one wife (OR = 3.57, CI 95% 1.20–10.58, *p* value 0.02). Children with more disabilities had a higher probability of being out of school. These children were 3.99 times more likely to be out of school compared to those with at least one disability (OR = 3.99, CI 95% 1.95–8.12, *p* value 0.02). The interaction between children with disability and the wealth index quintile was not statistically significant for out of school. However, children with disabilities in the 4th quintile wealth index were less likely to be out of school (OR = 0.23, CI 95% 0.06–0.87, *p* value 0.03).

**Table 3:** Binary logistic model predicting disable children out of school.

Variable	OR	(95% CI) LL—UL	Ρ value
Age of child	1.34	1.18–1.52	0.00
Disable children sex (Female as reference)			
Male	8.47	4.06–17.67	0.00
Total number of children living in this house (1 to 4 children as reference)			
5 and more children	5.71	1.03–31.58	0.05
Household head sex (Male as reference)			
Female	4.70	1.76–12.52	0.00
Education of household head (Educated as reference)			
Preprimary or none	4.29	1.91–9.57	0.00
Mothers Education (Educated as reference)			
Preprimary or none	1.20	0.58–2.48	0.62
Area (Urban/Rural) (Urban as reference)			
Rural	0.07	0.02–0.18	0.00
Husband age (Less than or equal to 50)			
51 years and above	2.64	1.37–5.12	0.00
Husband has more wives (No as reference)			
Yes	3.57	1.20–10.58	0.02
Disability types (at least one disability as reference)			
More disability	3.99	1.95–8.12	0.00
Wealth index quintile (Richest (5th) quintile as reference)			
1st quintile	2.83	0.79–10.16	0.11
2nd quintile	2.28	0.66–7.81	0.19
3rd quintile	1.94	0.60–6.23	0.27
4th quintile	0.23	0.06–0.87	0.03

Note: OR, Odds ratio; CI, Confidence interval; LL, Lower limit; UL, Upper limit; Significance at the 0.05 level.

Base choice for outcome variable is Disable children in school.

## Discussion

The overall aim of this study is to measure the association between disabled children out of school and different socio-economic factors in Bangladesh among the age group of 6 to 17 years of age. In developing countries like Bangladesh, disabled children are facing barriers in obtaining education although there are enough laws, policies, and services for them. Inadequate institutional services, weak academic assistance, lack of trained teachers and poverty are major causes of disabled children’s higher vulnerability to out of school. However, the underlying causes remain hidden in our social system [10, 15]. We found that an alarmingly high proportion of disabled children in Bangladesh are out of school. As a result, older children were more likely to be out of school than younger children To understand whether this association holds across different age groups, we conducted an additional analysis of the independent variables’ associations with school attendance across various age groups. Our findings indicate that the impact of factors such as household income, parental education, and geographical location on school attendance is consistent across all age groups. However, the magnitude of these effects varies, with older children experiencing more significant barriers. Furthermore, while education policy certainly plays a role in this phenomenon, it is essential to consider other contributing factors. Some scholars argue that economic distribution and poverty significantly impact school attendance for older children [15]. In many cases, older children from economically disadvantaged families may be required to work to support their households, leading to decreased school attendance. This perspective highlights the importance of addressing economic barriers to improve educational outcomes for older children.

Integrating both perspectives, our discussion acknowledges that while education policies are crucial, addressing economic challenges and poverty is equally important. Policies aimed at reducing the economic burden on families, such as providing financial incentives for school attendance or implementing programs that support the economic needs of families with school-aged children, could be effective in increasing school attendance among older children.

It is possible to partially explain as many disabled children leave school early due to education policy. Often, they are restricted to formal education in many countries. These findings are consistent with previous studies [3, 22]. Disabled male children were found to be at a higher risk of being out of school than female children. Very few studies find similar results [20, 23]. In general, research in developing countries suggests that being female has a negative impact on school participation [24]. However, the results seem to be different in Bangladesh, where female disabled children are more likely to attend school. According to the Bangladesh Demographic and Health Survey 2007, only 77.4% of male children were found attending school, compared to 81.9% of female children [25]. This trend of higher school attendance among female students in Bangladesh can be attributed to several factors.

Firstly, numerous initiatives by stakeholders have been implemented to promote school attendance among female students. These initiatives include offering free education up to the secondary school level, providing stipends and scholarships for female students, and implementing programs aimed at raising awareness about the importance of girls’ education. These efforts have collectively contributed to encouraging parents to send their daughters to school.

The effectiveness of these initiatives is also supported by other studies. For example [15] discusses how such policies have positively impacted female school attendance rates in Bangladesh. According to this reference, ’the concerted efforts to promote female education through various government and non-government programs have significantly improved school attendance rates among girls.’

In addition, cultural shifts and increased awareness about the value of educating girls have played a crucial role. The growing recognition of the long-term benefits of female education, including economic empowerment and improved health outcomes, has also encouraged families to prioritize education for their daughters.

In conclusion, while our findings on female school attendance align with the broader context of educational initiatives and cultural changes in Bangladesh, it is important to give due credit to the existing literature that supports these observations. By acknowledging these sources, we provide a comprehensive understanding of the factors contributing to higher school attendance among female disabled children in Bangladesh.

In addition, results showed that disabled children were more likely to be out of school if the household head was female. According to various studies, female-headed families in Bangladesh suffer more from poverty and food insecurity than male-headed families [20, 26, 27]. As a result, female-headed households face greater time and financial constraints that they are unable to overcome because their resource allocation is less child-centered and their children have less access to social services such as education and health care than children in male-headed households [26, 27]. The findings clearly show that poverty and female headship are strongly related. Thus, female headship could be a useful targeting indicator for poverty reduction in Bangladesh.

The results showed that disabled children with an uneducated household head had a higher probability of being out of school as compared to those who had an educated household head. Basically, the causal relationship between disability and being out of school is multidimensional and includes multiple factors rather than being a simple linear relationship. Several studies have indicated that the educational level of household heads has an impact on their children’s educational attainment as well as their decision to send their children to school [15, 28, 29]. Disabled children whose parents were educated appeared to be significantly more positive about school attendance [15]. These results indicate that there is a need to develop more strategies for households with disabled children. Additionally, rural disabled children are more likely to be out of school as compared to urban ones While our initial findings, as presented in Table 2, indicate that rural disabled children have similar or even higher school attendance rates compared to their urban counterparts, this contradicts our earlier assertion. This discrepancy aligns more closely with the findings of another study [30]. Karim et al. reported higher school attendance rates among rural children in Bangladesh.

Lamichhane and Kawakatsu [15] provide a possible explanation for this phenomenon, highlighting the positive effect of extended family support in rural areas. In rural settings, extended families often play a crucial role in supporting children’s education, which can lead to higher attendance rates. Additionally, previous reports from the Bangladesh Demographic and Health Survey (BDHS) have demonstrated higher attendance rates among rural children.

To clarify this discrepancy, it is important to consider the multifaceted factors that influence school attendance. In rural areas, the presence of extended family support, community-based educational initiatives, and possibly fewer economic opportunities for child labor might contribute to higher school attendance rates. On the other hand, urban areas, despite having better access to educational facilities, might face challenges such as higher living costs and the necessity for children to contribute to household income, potentially leading to lower school attendance rates.

Therefore, our findings suggest that while rural disabled children face significant challenges, the support structures in place may mitigate some of these barriers, resulting in relatively higher school attendance rates. This insight underscores the importance of tailored policy interventions that consider the unique socio-economic contexts of rural and urban areas to effectively address the educational needs of disabled children.

In Bangladesh, disabled children in rural areas are considered more vulnerable to accessing education due to poverty, the availability of special education systems and transportation as these results are supported by the studies [20, 31, 32]. Children’s services are concentrated in larger cities; therefore, students in more rural regions are underserved due to a lack of resources and teacher training [20]. School enrollment should be improved in rural areas by making special schools with specially trained teachers, including building ramps, more space in classrooms, height of the bench, wide doorways, and accessible toilets. Additionally, transport facilities from the rural areas to the urban areas should have adequate transport facilities for disabled children. That will ultimately reduce the dropout rate. Results show that there is a significant relationship between a husband’s age and his disabled children’s schooling status. Regarding the husband’s age, it indicates that children with an aged father (51 years and above) have a greater probability of being out of school. The lack of the husband’s involvement to accompany the child on school trips was recognized as an issue for out-of-school disabled children. Disabled children who are out of school having a father with an older age could indicate that a family’s human resources play an important role in supporting disabled children’s involvement in school [33, 34]. Those familial human resources could be better supported in order for all children to be included in the educational system by public and community resources and processes. For example, to form commuter groups or organize group transportation, the community could mobilize resources. Government assistance is required to assist families in gaining access to schools where disabled children were previously unable to attend.

The out of school rate was higher among those children whose disabled mother’s husband had more than one wife. Several studies have found that when it comes to supporting disabled children, one’s spouse is the best predictor of parenting quality. Parents may have a higher level of depression by having a child with disabilities that ultimately affects their relationship and disrupts family life [35–37]. Extramarital affairs by the father of a disabled person are assumed to be less responsible for the child [35] Additionally, the presence of multiple wives in a household can negatively impact the school attendance of disabled children [35]. This phenomenon can be explained by the distribution of household resources and attention among multiple wives and their children, which may result in reduced support for each child. While specific studies from a similar cultural context are limited, this observation aligns with general findings on the impact of polygamous households on children’s education. Further research is needed to explore this dynamic in greater detail within South Asian rural contexts. As a result, this disables children’s mothers who do not get support from family and community. So, government and non-government organizations have to come up with ideas to support disabled children’s mothers. Having more disabilities is a strong risk factor for being out of school [15, 29, 34]. The barrier to school is higher because of a combination of multiple disabilities such as visible, physical, learning and so on [3, 15, 29]. Due to various types of disability, students may face barriers if the school is not within their reach zone and the accessibility and availability of public transport [38]. Furthermore, children with multiple disabilities were less likely to attend school due to discrimination and barriers from both families and institutions [15, 28]. it is urgent to provide additional school opportunities for children with more disabilities, taking into account family perceptions. Disability is a significant reason for being absent from school. Improvement of the school environment by special trained teachers could enhance the school’s accessibility. Although many basic components are covered by the government’s policies and plans, there is a scarcity of implementation at school and in the family. In Bangladesh, the government, NGO’s and education departments should coordinate funding for the improvement of disability schooling status. Social workers, educational officers, and other stakeholders must maintain regular contact to properly implement the plans by the school staff and parents.

However, several limitations exist in our study. First, we cannot identify the causal factors explaining why disabled children are out of school because the analysis is based on cross-sectional data. It can only explain the association. Second, the information about disability given by the mother or caretaker may be biased. It’s also important to remember that disability and poverty are found to be positively linked in many studies, and that being out of school can be a result of both poverty and disability. Poverty can have a negative impact on creating disabilities and school enrollment, and the study is unable to differentiate between the two. A longitudinal study could be useful to address the above-mentioned limitation through routine disability reporting with wealth inequality. Further research will also seek to understand why the policy regarding disability education was not implemented properly. Poverty, teacher shortages, the learning process, and a lack of planning may be the primary causes of policy implications for disability education.

Despite the limitations mentioned above, the current study provides unique data on out of school among Bangladeshi disabled children. The results can be helpful in understanding the associated factors that keep disabled children out of school and can provide valuable insights for better policy formulation aimed at improving school attendance among disabled children. However, the current policy regime in Bangladesh, including the National Education Policy, the Disability Rights and Protection Act, and initiatives under the Ministry of Primary and Mass Education (MoPME), faces significant limitations such as inconsistent implementation, lack of awareness and advocacy, socio-economic barriers, and insufficient monitoring and evaluation. To address these issues, we recommend strengthening the implementation and resource allocation to ensure schools have the necessary infrastructure and trained teachers, enhancing awareness and advocacy programs to raise the importance of education for disabled children, providing targeted financial support and incentives to families, especially in rural areas, and developing comprehensive training programs for teachers on inclusive education practices. Additionally, establishing robust monitoring and evaluation mechanisms and revising specific policies to include continuous professional development of teachers and stronger enforcement mechanisms can create a more inclusive and supportive educational environment. By bridging the gap between policy intentions and on-the-ground realities, these targeted reforms can improve school attendance and educational outcomes for disabled children in Bangladesh.

We need to know what services are required for disabled children, where they should be provided, and how much they will cost. We also need to know how much educational services would motivate families to take their children to school and decrease the stigma associated with disabilities. Despite constitutional guarantees and legal protections, disabled children in Bangladesh encounter a variety of difficulties and limitations when it comes to obtaining an education. The study could be one of the best attempts to address the rights of people with disabilities.

Our findings suggest that to improve school attendance for older children, policymakers should consider both educational and economic interventions. While enhancing education policies to make schooling more accessible and appealing is vital, addressing the economic needs of families is equally crucial. Providing support such as scholarships, free school meals, and after-school programs could alleviate some of the economic pressures that force older children to leave school.

By addressing both educational and economic factors, we can create a more holistic approach to improving school attendance for children with disabilities in Bangladesh.

## Conclusion

Using nationally representative data, the current study primarily focused on the schooling status of disabled children and examined the factors that contributed to their absence from school. Overall, in Bangladesh, 22.6% of disabled children are out of school. Binary logistic regression was used to find out the associated factors for disabled children out of school among the age group of 6 to 17 years. Our findings indicate that the age of the child, gender, household head sex, education of the household head, area (urban/rural), husband age, husband having more wives, and disability types are associated factors for disabled children being absent from school. This study’s variables are similar to those employed by Lamichhane and Kawakatsu (2015) [15], who also investigated the determinants of school attendance using variables such as religion, work in agriculture, the number of working-age population, and monthly household expenditure.2 Despite the overlap in variables, our study adds to the existing knowledge in several keyways.

Firstly, while Lamichhane and Kawakatsu [15] did not find a significant urban/rural divide in school attendance, our findings highlight the unique challenges and support systems present in rural areas, suggesting that rural disabled children have higher school attendance rates compared to their urban counterparts. This discrepancy underscores the importance of context-specific interventions and the need to tailor policies to address the unique socio-economic dynamics of different areas.

Additionally, our study introduces the variable of the husband’s age and the number of wives, which, although not previously considered, sheds light on the complex family dynamics that can influence a child’s education. This novel insight can inform more nuanced policy formulations that consider family structures and their impact on children’s schooling.

Furthermore, our findings reinforce the significant role of household head education in promoting school attendance among disabled children. This aligns with Lamichhane and Kawakatsu’s [15] conclusions, but our study further emphasizes the critical need for educational policies that support not only the children but also aim to educate and empower the household heads.

Our study’s contribution lies in its detailed exploration of the intersection between disability and educational access, providing specific insights into how different factors interact to influence school attendance. This comprehensive understanding can better inform policymakers in crafting targeted interventions that address the multifaceted barriers faced by disabled children.

In conclusion, while our findings align with much of the existing literature, they also offer new perspectives and emphasize the need for targeted, context-specific policies. By acknowledging the similarities and differences with previous studies, we contribute to a more nuanced understanding of the factors affecting school attendance for disabled children, ultimately aiding in the formulation and execution of more effective educational policies.

To overcome this issue, there is a need for a clear policy implication for disabled children’s education. One of the most effective ways of encouraging disabled children to attend school would be the reduction of tuition fees and providing transportation assistance. A program should be implemented to raise awareness among parents, which ultimately encourages parents of disabled children to more actively invest in their education. As well, any societal, institutional, or financial barriers that limit disabled children or prevent them from exercising their right to education should be removed. The government should take a more active role in enforcing current laws, regulations, and measures that protect the educational rights of disabled children. Policymakers and other stakeholders should take immediate action to protect disabled children’s educational rights so that they can face fewer difficulties in their daily life.
